# Reflecting on the 1998 enterovirus outbreak: A 25-year retrospective and learned lessons

**DOI:** 10.1016/j.bj.2024.100715

**Published:** 2024-03-15

**Authors:** Peng-Nien Huang, Shao-Hsuan Hsia, Kuan-Ying Arthur Huang, Chih-Jung Chen, En-Tzu Wang, Shin-Ru Shih, Tzou-Yien Lin

**Affiliations:** a Research Center for Emerging Viral Infections, Chang Gung University, Taoyuan, Taiwan; b Division of Infectious Diseases, Department of Pediatrics, Chang Gung Memorial Hospital, Linkou, Taoyuan, Taiwan; c Division of Pediatric Critical Care Medicine, Department of Pediatrics, Department of Pediatric Respiratory Therapy, Chang Gung Memorial Hospital, Linkou, Taoyuan, Taiwan; d College of Medicine, Chang Gung University, Taoyuan, Taiwan; e Division of Acute Infectious Diseases, Centers for Disease Control, Ministry of Health and Welfare, Taiwan; f Department of Medical Biotechnology and Laboratory Science, College of Medicine, Chang Gung University, Taoyuan, Taiwan; g Department of Laboratory Medicine, Chang Gung Memorial Hospital, Linkou, Taoyuan, Taiwan; h Research Center for Chinese Herbal Medicine, Research Center for Food and Cosmetic Safety, and Graduate Institute of Health Industry Technology, College of Human Ecology, Chang Gung University of Science and Technology, Taoyuan, Taiwan; i Department of Pediatrics, Chang Gung Memorial Hospital, Linkou, Taoyuan, Taiwan; j Department of Pediatrics, College of Medicine, Chang Gung University, Taoyuan, Taiwan

## Abstract

Enterovirus A71 (EV-A71) infections are a major Asia-Pacific health issue. However, this infection can cause serious and potentially fatal neurological issues. We attempt to explain EV-A71's molecular virology, epidemiology, and recombination events in this review. The clinical and neurological signs of EV-A71 infections are well documented. The review discusses EV-A71 central nervous system infections' causes, diagnostic criteria, treatment choices, and prognosis. Some consequences are aseptic meningitis, acute flaccid paralysis, and acute transverse myelitis. These problems' pathophysiology and EV-A71's central nervous system molecular processes are examined in the review. EV-A71 infections must be diagnosed accurately for therapy. No particular antiviral medications exist for EV-A71 infections, thus supportive care is the main treatment. The study emphasises addressing symptoms including temperature, dehydration, and pain to ease suffering. EV-A71 CNS infections have different prognoses depending on severity. The review discusses long-term effects and neurological sequelae of EV-A71 infections. In conclusion, Asia-Pacific public health is threatened by EV-A71 infections. This review helps prevent, diagnose, and treat EV-A71 infections by addressing the mechanisms, diagnostic criteria, treatment choices, and prognosis. This study fully examines the challenges and considerations of managing and treating EV-A71 infections. It also recommends future research and development to generate effective viral infection treatments.

## Highlights

• We examine EV-A71's virology, epidemiology, and recombination events.
• The review covers clinical features, diagnosis, treatment, and prognosis of EV-A71 infections.
• The paper compiles global EV-A71 and HFMD data and discusses vaccine development progress.

## 1 Introduction

Emerging virus status is assigned to Enterovirus A71 (EV-A71), which has the potential to cause future epidemics on a significant scale. As the virus continues to evolve, there is a risk of increased transmission and the potential for outbreaks. Developing strategies for early detection, effective treatment, and preventive measures is crucial to mitigating the impact of EV-A71 on public health. A comprehensive review of the current understanding of EV-A71, its pathogenesis, and the existing preventive and therapeutic approaches would be essential. Additionally, exploring the potential future trends and challenges associated with EV-A71 infections can contribute to informing public health policies and preparedness for emerging viral threats.

Hand, foot, and mouth disease (HFMD) is typically a mild childhood disease characterized by fever and skin rash, caused by human enteroviruses. In developing areas, the route of transmission is believed to be primarily through the ingestion of contaminated fecal matter, while in developed areas, it is attributed more to respiratory droplets. The transmission of HFMD is facilitated by the ability of these viruses to survive for extended periods in the environment and their higher resistance to disinfectants [1]. Human enterovirus (HEV) members EV-A71, coxsackievirus A6 (CVA6), CVA10, and CVA16 are the pathogenic viral agents accountable for this illness. CVA16 and EV-A71 are widely acknowledged as the principal causative agents of this prevalent ailment in children across the globe. Infections brought on by the EV-A71 have been linked to significant negative effects on public health in the Asia-Pacific region [2]. The frequency of a number of different illnesses, with one particular pathogen being one of the primary responsible parties. This virus is responsible for causing severe neurological problems in some people with HFMD, which can sometimes lead to catastrophic consequences. The EV-A71 virus is a member of the *Picornaviridae* family and consists of a single molecule of positive-stranded RNA that is approximately 7500 nucleotides in length [3,4]. The RNA genome of the virus encodes a polyprotein that is all-encompassing. This polyprotein is made up of structural proteins (VP4, VP3, VP2, and VP1) as well as nonstructural proteins (2A, 2B, 2C, 3A, 3B, 3C, and 3D).

The molecular virology, epidemiology, and recombination events linked with EV-A71 are investigated in depth throughout this comprehensive analysis. The review also investigates a full examination of the clinical characteristics and neurological symptoms connected to EV-A71 infection, with a specific emphasis on brainstem encephalitis as the primary focus of the investigation. In addition to this, the review investigates the neurological complications that are caused by an infection with EV-A71 that affects the central nervous system (CNS). Additionally, the review investigates the underlying pathophysiology, diagnostic tools, treatment choices, and prognosis for this condition. In conclusion, this study examines and assesses the developments that have taken place in the sector of innovative vaccine development approaches.

## 2 Molecular virology

Multiple viral receptors, such as human scavenger receptor B2 (hSCARB2), human P-selectin glycoprotein ligand 1 (PSGL-1), dendritic cell-specific intercellular adhesion molecule-3 grabbing nonintegrin, annexin A2, heparan sulfate, and sialylated glycan, have been found to facilitate the entry of EV-A71 into host cells [[5], [6], [7], [8], [9], [10]]. The hSCARB2 receptor was first found to be an EV-A71 receptor on rhabdomyosarcoma cells. Since then, it has been found to demonstrate expression in a variety of cell types, including neurons in the central nervous system. This expression pattern suggests that hSCARB2 may play a role in enabling EV-A71's direct infection of the brain. During the process of leukocyte infiltration, leukocytes express the protein PSGL-1, which is thought to play a function in the initial attachment and migration of leukocytes along the vascular endothelium. PSGL-1 is thought to be similar to sialomucin and is expressed in leukocytes. Previous research [6] has demonstrated that EV-A71 is able to successfully infect T cells when those T cells express PSGL-1.

After the viral particle has entered the host cells, it is important to note that, in stark contrast to the messenger ribonucleic acids (mRNAs) that are found in eukaryotic organisms, EV-A71 possesses an internal ribosome entry site (IRES) structure located at the 5′ end of its viral genome. This IRES structure allows the virus to replicate by allowing ribosomes to enter the viral genome. The structure of the IRES is of critical significance in terms of easing the process of viral translation since it permits the translation of viral RNA via a mechanism that is IRES-dependent. At the true initiation codon, the process of initiation takes place. At this point, the 40S ribosomal subunit detects a particular sequence, RNA structure, or ribonucleoprotein complex that is positioned within the IRES. It has been discovered that a number of *trans*-acting factors, which are collectively referred to as IRES-specific *trans*-acting factors (ITAFs), can have a constructive effect on the regulation of the activity of an IRES. These ITAFs consist of heterogeneous nuclear ribonucleoprotein A1, heterogeneous nuclear ribonucleoprotein K, far upstream element-binding protein 1, T-cell-restricted intracellular antigen 1 (TIA-1), and TIA-1-related protein. It is well established that these factors attach themselves with the 5′ end of the EV-A71 viral genome and participate in either the replication of viral RNA or the activation of viral IRES. The extra cellular protein known as far upstream element-binding protein 2 (FBP2) has the role of acting as a suppressor of the activity of the IRES [[11], [12], [13], [14], [15], [16]]. The reduction of IRES-mediated EV-A71 translation in infected cells is caused by the interaction between FBP2 and the Kelch domain of Kelch-like protein 12, as well as the involvement of the C-terminal domain of FBP2 in FBP2 ubiquitination. Both of these interactions take place on the same protein, FBP2. Because of this interaction, the competitive advantage enjoyed by FBP2 in comparison to other positive ITAFs is enhanced [17].

## 3 Clinical epidemiology

**Fig. 1:**
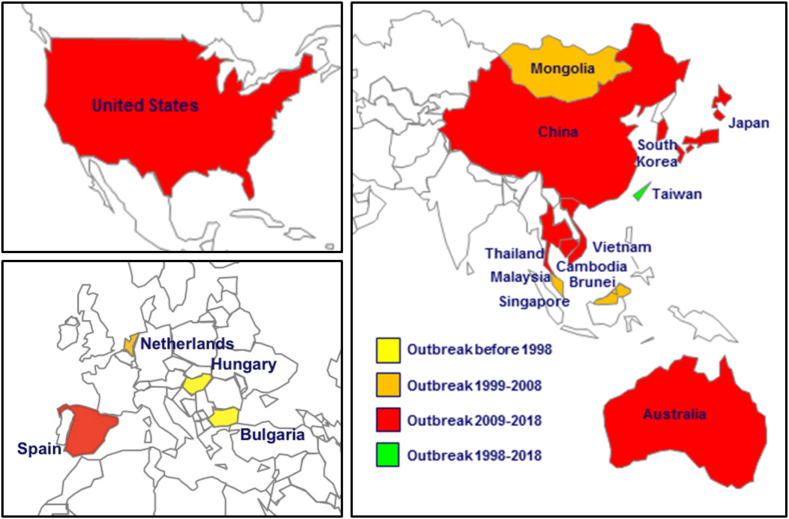
Countries with reported EV-A71 epidemics, 1998–2018.

**Fig. 2:**
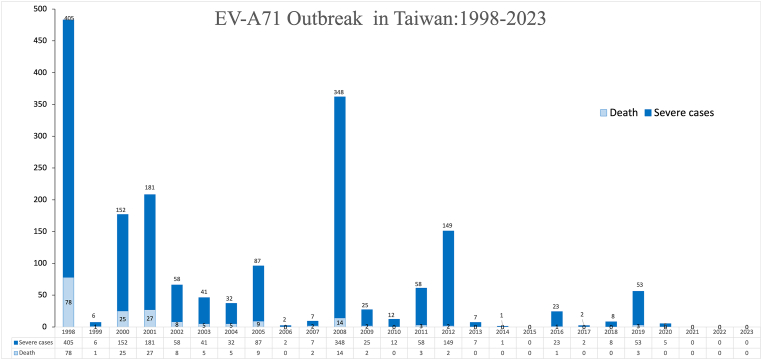
The dataset represents the annual enumeration of severe EV-A71 cases and consequent fatalities in Taiwan from the period spanning 1998 to 2018. Severe instances are defined as those that have, at any time, been classified as World Health Organization (WHO) Stage II (with Central Nervous System, CNS, involvement) or higher.

In 1969, the first report on the isolation of EV-A71 was published. At that time, the virus had been taken from the brain of an infant born in the United States who was suffering from encephalitis. HFMD is a condition that can occur anywhere in the world. However, since 1997, there has been a distinct pattern of rising HFMD outbreaks in the Asia-Pacific region (see [Table 1], [Fig. 1]). In the middle of the year 1997, there were multiple reports of children in the Malaysian state of Sarawak losing their lives. The patients who passed away presented with signs of a febrile illness that moved quickly through the stages of cardiopulmonary failure. On the other hand, the fundamental cause of this illness has not yet been determined [18]. In 1998, Taiwan was hit by an outbreak of HFMD, which led to a total of 405 severe cases of neurological sequelae, such as meningitis and encephalitis. The disease was first identified in the United States in 1999. Unfortunately, as a direct consequence of this outbreak, 78 people did not make it through the ordeal alive [3,19]. The HFMD which was caused by EV-A71, was responsible for 387 severe cases and 14 fatalities in Taiwan in 2008 [20]. This was ten years after the disease was first identified. Information on the number of severe cases and deaths may be found in the data that was collected in Taiwan on a yearly basis from 1998 to 2017. For the purposes of this investigation, severe cases were defined as those cases that fell under the stage II or higher classification of the World Health Organization (WHO), specifically cases including involvement of the CNS. Prior to the year 2012, there was a pattern of recurrent large-scale outbreaks occurring every two to four years. This pattern could possibly be ascribed to the growing population of young children who were not vaccinated, as this population was rising at the time. Notwithstanding this, there has been a reduction in the prevalence of severe cases since the year 2012 (see [Fig. 2]), which may be attributable to advancements in public health infrastructure, the introduction of multidisciplinary pediatric care, or the occurrence of virus recombination.

**Table 1:** HFMD outbreaks and severe and fatal cases, 1997–2018.

Year	Location	HFMD Case Number	Severe Case Number	Fatal Case Number
1997	Malaysia	2628	889	29
1998	Taiwan	129,106	405	78
2000	Japan	205,365	272	1
2000	Singapore	3790	–	3
2001	Singapore	5187	–	3
2000–2002	Taiwan	–	844	129
2003	Japan	172,659	–	–
2005	Taiwan	–	142	16
2005–2007	Vietnam	1069	87	6
2006	Brunei Darussalam	1681	–	3
2008	China	488,955	–	126
2008	Mongolia	3210	–	–
2008	Taiwan	–	387	14
2008–2009	Vietnam	27,309	–	71
2008–2012	China	7,200,092	–	2452
2011–2012	Vietnam	200,000	–	207
2012	Taiwan	–	153	2
2013	Japan	297,879	–	–
2014	China	2,712,925	–	384
2016	China	2,468,174	–	220
2018	Colorado, USA	–	34	0

**-,** No data available.

In the year 1981, the HFMD was identified for the very first time in the city of Shanghai in China. A total of 488,955 instances of HFMD were reported in the year 2008, and 126 people lost their lives as a result of the illness. According to these numbers, HFMD has undergone a dramatic transformation, developing into a dangerous illness and becoming a primary contributor to the mortality rate of children [21,22]. 2003 marked the first year that EV-A71-associated HFMD was documented as having occurred in Vietnam. After that, in 2011 and 2012, there was a considerable increase in the number of cases of HFMD, which surpassed 200,000 and led to 207 fatalities [23]. 2009 was the year when South Korea reported its first incidence of HFMD, which sadly resulted in a fatality [24]. Some countries in the Asia-Pacific region, such as Japan (in 1997 and 2000) and Singapore (in 2000), continue to struggle with HFMD epidemics as a frequent problem [25]. As of the 26th of August in 2018, it was discovered that EV-A71, a virus that is related with neurological diseases [26], has impacted a total of 34 children in the state of Colorado, which is located in the United States. It is also evident that outbreaks of HFMD are not restricted to countries in the Asia-Pacific region, but are also detected in European and American regions, including Spain and the United States. This is the case since HFMD is a highly contagious disease that may spread easily from person to person.

## 4 EV-A71 genotype and disease severity

Based on the sequencing of the VP1 gene, EV-A71 can be broken down into four primary genotypes, designated A through D. Both the B and C genotypes can be further classified into a total of ten different subgenotypes, designated as B1–B5 and C1–C5, respectively [27,28]. An exhaustive study conducted between 1963 and 1967 on EV-A71 strains that originated in the Netherlands uncovered the presence of a distinct subgenotype B0 for EV-A71 [29]. This discovery was made possible by the fact that the Netherlands was the only country in which EV-A71 was found. During the course of an in-depth examination of the genomes of EV-A71 strains [30,31], a distinction was made between the genotype D of EV-A71 and the subgenotype C4 of that strain. In recent years, it was discovered that genotype D was present in India, while in Africa, novel genotypes E and F were recognized [32,33]. Both of these discoveries occurred very recently. It is most common to find EV-A71 with the genotype C4 in China, Korea, and Vietnam; on the other hand, genotype B5 is frequently found in Taiwan, Japan, and Malaysia.

Numerous pieces of research have shed light on the relationship that exists between EV-A71 genotypes and the manifestations of the disease. In a study that was carried out in Australia in 1999 [34], a connection between severe neurological disease and EV-A71 of the subgenotype C2 was detected. Infections induced by the EV-A71 subgenotype B4 in Malaysia were found to have a decreased risk of resulting in CNS sequelae when compared to infections generated by other subgenotypes [35]. This was observed in comparison to infections generated by other subgenotypes. It has been hypothesized, on the basis of research carried out by Dutch specialists, that children who are infected with genotype B of the EV-A71 virus are more likely to experience neurological abnormalities compared to those who are infected with genotype C [29]. This hypothesis is supported by the findings of the research. Between the years 1998 and 2000, research that was carried out in Taiwan indicated that genotypes B and C of EV-A71 were detected in instances of HFMD that ranged in severity from deadly to mild [36,37]. In mouse infection models, it was observed that EV-A71 subgenotype C4a was significantly more severe than EV-A71 subgenotype B5 [38]. When the whole genomes of EV-A71 bacteria collected from mild cases and those recovered from severe cases were compared, it was shown that the nucleotide changes in the latter were largely situated inside the protein-coding area. This was discovered by comparing the two sets of EV-A71 strains. It is important to point out that a number of highly pathogenic strains have been found to contain an amino acid substitution in which glutamine (Q) replaces glutamic acid (E) in the 145th position of the VP1 structural protein. This substitution has been observed. The previously described website has been credited with having a key role in receptor binding and pathogenicity in mice [39], as this was previously mentioned. For this reason, it is essential to carry out a comprehensive study in order to gain a better understanding of the connection that exists between the various genotypes of EV-A71 and the degree of severity of the disease. It is likely easier to see sudden changes in the dominance of different EVA-71 genogroups or subgenogroups in the same country or area because different lineages are always moving around and switching who is in charge. In addition, sequence recombination and spontaneous mutations in the viral genome increase genetic diversity and drive the evolution of EV-A71 strains.

## 5 Recombination events in EV-A71

The presence of many viruses within a single cell has the ability to stimulate recombination processes, which can result in the creation of new genomic configurations in a virus [40]. This has the potential to lead to the development of new infectious diseases. Recombination events that take place in RNA viruses have the ability to increase their pathogenicity as well as widen their spectrum of hosts that they can infect. Enteroviruses are known to undergo recombination events on a regular basis, and these events tend to take place at recombination sites that are situated inside the nonstructural protein-coding regions P2 and P3. These regions have a substantial degree of similarity in the nucleotide sequences of both of the parental strains, which in turn encourages the occurrence of homologous recombination [41,42]. When doing recombination studies, researchers usually look for breakpoints in the 5′ untranslated region (5′UTR), P2, and P3 by sequencing either distinct gene parts or the entire genome [30,43,44]. In a study that was carried out by experts, a total of 11 unique clades of viral 3D protein sequences were revealed in EV-A71 isolates that were taken from 19 countries that are geographically distinct from one another. Each of these clades displayed a selectivity toward EV-A71 and was associated with a certain subgenotype. On the other hand [45], research indicated that they were phylogenetically intermixed with clades of CVA16 as well as other serotypes that belonged to the HEV-A genus.

Since 1998, Taiwan has been the scene of many outbreaks, all of which have been traced back to various recombinant strains of the EV-A71 virus. One illustration of this phenomenon is the emergence of the EV-A71 subgenotype C2 as a result of intertypic recombination between the EV-A71 virus and the coxsackievirus A8 virus. In the year 1998, a notable outbreak was brought on by a particular subgenotype that was responsible [20,46]. It is believed that subgenotype B4 of the EV-A71 virus, which caused the ensuing epidemics in the years 2000 and 2001, originated from subgenotypes B3 and B2 of the virus. It is possible that intratypic recombination between genotype C and genotype B was responsible for the appearance of the dominant EV-A71 subgenotype C4, which took place between 2004 and 2005 [20].

The recombination of EV-A71 and Coxsackievirus A16 (CVA16) was suspected to be the cause of a considerable number of cases of HFMD in China in the years 2007 and 2008. These years saw an outbreak of HFMD in China. An examination of the complete genomes of the EV-A71 and CVA16 strains received from Shenzhen revealed evidence of the presence of intertypic recombination at the 2A–2B junction of a CVA16 strain, which involved the EV-A71 genotype A [30]. The research found instances of double recombinants in China, where intratypic recombination took place between genotypes C and B of EV-A71, and where intertypic recombination took place between genotype B of EV-A71 and CVA16 [30]. Based on the findings of this investigation, a reclassification of the EV-A71 subgenotype C4 as the recently discovered genotype D is proposed. When compared to subgenotypes C1 to C5, it was found that the C4 virus subtype had a larger nucleotide sequence divergence, which ranged from 17% to 20%. The specific connection that exists between the spontaneous recombination that occurs in EV-A71 and the pathogenicity of the virus is not yet completely understood. An experiment carried out in a laboratory, on the other hand, has shown evidence suggesting that an artificially produced recombinant virus with accelerated proliferation and larger plaque morphologies is possible. To accomplish this, the structural section of a slow-growth EV-A71 strain was replaced with the same region from a rapid-growth EV-A71 strain [47]. This resulted in the strain having quick growth. Therefore, it is possible for a strain with a lower infectious potential to experience spontaneous recombination, which would result in the acquisition of an antigenically distinct capsid area or nonstructural parts from a strain with a higher infectious potential. This mechanism has the potential to one day result in the production of a strain of EV-A71 that is particularly dangerous to humans.

## 6 Clinical manifestation and management

On initial evaluation, around ninety percent of patients were found to have symptoms of herpangina in addition to those of HFMD. This was determined by observing the patients' medical histories. Additional clinical signs, such as nonspecific febrile illness, upper respiratory tract infection, enteritis, and viral exanthema, are reported in these patients a lower percentage of the time [48,49].

HFMD disease is associated with a number of serious neurological complications, some of the most famous of which are aseptic meningitis, encephalitis, acute flaccid paralysis, encephalomyelitis, and brainstem encephalitis. It is important to point out that brainstem encephalitis is the most severe manifestation of progressive HFMD linked with EV-A71. This is one of the reasons why this condition is so dangerous. During the HFMD epidemic that occurred in Taiwan in 1998, a comprehensive study of the clinical presentations of HFMD cases that included the CNS revealed that recurrent myoclonus was the most common neurological explanation. In addition, there is a possibility that additional symptoms, such as tremors, ataxia, and cranial nerve palsies, will be present. Children who are most seriously affected may suffer from autonomic dysfunction, neurogenic pulmonary edema, neurologic shocked myocardium (as referred by Ref. [50]), or norepinephrine cardiotoxicity (as cited by Ref. [51]) [50,51]. are both sources of information. Even with the administration of contemporary intensive care assistance, the presence of these circumstances has the potential to trigger a swift progression toward fulminant cardiac failure, which can result in either death or a significant occurrence of severe neurological and psych behavioral complications among those who survive [52].

**Fig. 3:**
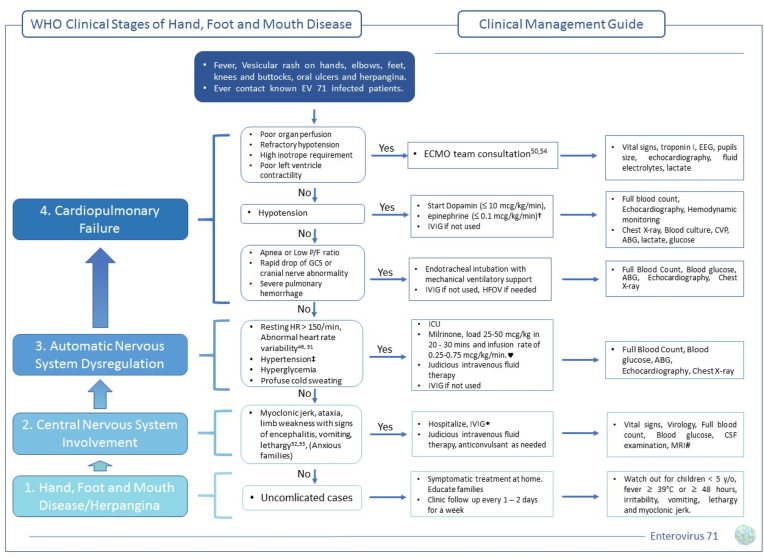
Clinical Manifestations and Management Strategies of Enterovirus Infections: An Examination of the Four-Stage Progression [[53], [54], [55], [56], [57]]. †Catecholamines have been observed to potentially induce neurogenic pulmonary edema, inflict damage to the myocardium, and enhance viral infection. Consequently, the World Health Organization guidelines [[58], [59], [60]] advise against the use of dopamine and epinephrine. However, it is crucial to note that these adverse effects are predominantly associated with the α1-adrenergic effect, which is prominent at high infusion rates of dopamine, epinephrine, and dobutamine [61]. Given that extracorporeal membrane oxygenation (ECMO) in infants and toddlers has been linked to severe complications [62], we still advocate for the administration of low to intermediate doses of dopamine and epinephrine prior to ECMO. ‡ Stage 1 hypertension is classified by systolic pressure that surpasses the 95th percentile for children of the same age, specifically 115 mmHg for high-risk age demographics, when in a calm state [63]. ♥ In relation to the application of milrinone, it was found that the 1-week mortality rate was lower, and the median duration of ventilator-free days was longer in the treatment group [64]. ∗Generally, a favorable prognosis is typically observed in patients diagnosed with aseptic meningitis; therefore, the use of intravenous immunoglobulin may not be necessary [48,65,66]. # Lastly, the use of computed tomography scan is not recommended in the management of these cases [2].

Cases of HFMD that are very severe often display a development of organ system involvement that manifests in four distinct clinical phases. People who have been through these terrible events might need to be cared for significantly longer periods of time. The clinical symptoms and recommended clinical care guidelines are outlined in a condensed format in [Fig. 3], which may be found here.

Unfortunately, a sizeable majority of people who have reached stage IV suffer from a variety of neurological repercussions, the severity of which can range from moderate to severe [67]. The evaluation of pulmonary function, the administration of chest physiotherapy, the provision of dietary assistance, and the implementation of rehabilitation measures are all included in the initial post-cessation care that is provided to survivors after smoking has been discontinued. These assessments are carried out with the assistance of magnetic resonance imaging (MRI) and electroencephalography (EEG). Patients who have limited or nonexistent spontaneous breathing and are unable to be properly weaned off the help of a mechanical ventilator are typically advised to go through with a tracheostomy procedure. This is the suggestion made by the medical community. According to the findings of a study that was carried out over an extended length of time, more than twenty percent of the people who survived developed neurological issues. These problems were of varying degrees of seriousness, ranging from mild limb weakness or atrophy to the requirement of support from a ventilator. Some people, when faced with extremely difficult circumstances, even entered a vegetative condition. It was noted that 18 of the patients who had survived cardiopulmonary failure had indications of limb weakness and atrophy. This represented 64% of the whole cohort of 28 patients who had survived the condition. In addition, 17 patients (61%) required nutrition through a tube, while 16 patients (57%) required assistance breathing through a ventilator. A study was conducted on patients who underwent the Denver Developmental Screening Test (DDST), which effectively assesses children's development by identifying potential delays in four key areas: personal-social competence, gross motor skills, fine motor skills, and language skills. Healthcare professionals rely on this test to promptly evaluate performance and identify any potential delays or the need for further evaluation. The findings of this study revealed a substantial proportion of individuals, specifically 75% (21 out of 28 patients), exhibited delayed neurodevelopment in the context of cardiopulmonary failure [67]. People who have successfully recovered from a medical condition are not the only kinds of people who can be considered to be survivors. These people have shown great resiliency by overcoming a complex and challenging health issue, and as a result, they have emerged on the other side with more strength and fortitude. On the other hand, the progression of their experience does not culminate in a state of recuperation being reached at any point in time. The people who survived the disaster require long-term support in many different areas, including medical care, financial assistance, and educational opportunities.

## 7 Development of EV-A71 vaccines

There has been a substantial amount of development in the field of EV-A71 vaccine research, with important breakthroughs emerging at a much faster rate. During the phase III clinical trials, three genotype C4 vaccines that were inactivated and assisted with adjuvants exhibited promising performance, and as a result, they were granted approval from the China Food and Drug Administration (refer to [Table 2]) [68]. Two clinical studies of phase III have been carried out in Taiwan to investigate the efficacy of inactivated genotype B4 vaccines in terms of both their safety and their ability to elicit an immune response,both Taiwanese vaccine manufacturers obtained Taiwan FDA Licensed in 2023." [Table 2] contains the findings obtained from the aforementioned experiments [68,69].

**Table 2:** Current stage of five adjuvanted and inactivated EV-A71 vaccines.

Developer	Strain (genotype)	Antigen amount	Cell line	Age^a^	Efficacy^b^	Status^c^
Chinese Academy of Medical Sciences, China	FY23 (C4)	100 U (2 μg)	Human diploid KMB-17	6–71 m	97.4%	Licensed in December 2015
Sinovac Biotech, China	H07 (C4)	400 U (1 μg)	Vero	6–35 m	94.8%	Licensed in January 2016
Beijing Vigoo Biological, China	FY7VP5/AH/CHN/2008 (C4)	320 U (0.5 μg)	Vero	6–35 m	90.0%	Licensed in March 2017
Enimmune, Taiwan	E59 (B4)	1 μg	Vero	2m-6y	ND	Licensed in February 2023
Medigen Vaccine Biologics, Taiwan	E59 (B4)	2.5 μg	Vero	2m-6y	ND	Licensed in April 2023

a Two doses of vaccines are administered intramuscularly 28 days apart.

b Efficacy against EV-A71-associated diseases is measured.

c Clinical trial registration: NCT01569581 (Chinese Academy of Medical Sciences, Phase III), NCT01507857 (Sinovac Biotech, Phase III), NCT01508247 (Beijing Vigoo Biological, Phase III), NCT01376479 (Inviragen, Phase I).

A group of 749 healthy newborns was studied as part of a prospective follow-up research that was carried out in Taiwan over the course of three years. The purpose of this study was to ascertain the prevalence of EV-A71 infection in infants who had not yet reached the age of 6 months, as well as in those who were between the ages of 13 and 24 months, and 25 and 36 months. According to the data, the age-specific incidence rate of EV-A71 infection in newborns younger than six months was 1.71 per 100 person-years. This rate was seen in infants younger than six months. This rate went up to 4.09 between the ages of 13 and 24 months, and then it went up even further to 4.97 between the ages of 25 and 36 months [70]. According to the findings of the epidemiological study, it is abundantly obvious that babies, particularly those younger than six months of age, are at the greatest risk of developing new EV-A71 infections. It has been shown that newborns are more prone to experiencing severe neurological disease and mortality during epidemics of EV-A71 [[71], [72], [73]]. This has been observed to be the case. The findings that were provided in this study have been corroborated by surveillance data gathered from other places that have been impacted by epidemics [[74], [75], [76]]. The utilization of the EV-A71 vaccine offers a potentially effective technique in managing epidemics and obtaining improved outcomes for vulnerable groups. Children aged two months to six years should be given top priority as the primary recipients of the benefits of the immunization policy, according to our argument, which advocates for this priority. As a significant step forward on the path toward the creation of vaccinations that combat infectious diseases, the development of an efficient vaccine against EV-A71 is an important accomplishment for pharmaceutical companies operating in Asia. The vaccine has a sizeable chance of lowering the incidence of the disease in geographical areas where it is common, such as the Asia-Pacific region, Europe, Australia, and the United States [26,73,77,78]. In order to determine the importance of the EV-A71 vaccine on a global scale, it is imperative to get data on the vaccine's efficacy by way of broad, randomized clinical studies carried out in a number of different countries and continents.

## 8 Future prospects for prevention and control of EV-A71 infection

The remarkable accomplishment of successfully eradicating poliovirus over the course of human history offers us a hopeful indicator that the prevention of EV-A71 infection and the management of its symptoms may soon be realizable. In spite of this, as we continue along this path, there are a number of roadblocks that we have to overcome in order to be successful in our effort to defeat EV-A71.

Even while it is true that inactive EV-A71 vaccines have showed safety, effectiveness, and the potential to produce powerful immune responses for protection against EV-A71 infection, it is vitally important to underline the significance of keeping tight production quality control standards. In this context, one of the most important things to do is to make certain that the effectiveness of vaccinations is maintained while still meeting all of the necessary safety standards. In order to ensure that the quality of vaccinations remains consistent throughout time, it is absolutely necessary to have monitoring procedures that are both comprehensive and ongoing. The challenge of developing EV-A71 vaccines with broad neutralizing activity stems from the virus's genetic diversity. EV-A71 has multiple genotypes and sub-genotypes, leading to variations in surface proteins. These proteins are the primary targets for neutralizing antibodies. Differences in key epitopes among genotypes can hinder the effectiveness of a vaccine designed for one strain. A vaccine with broad neutralizing activity requires addressing EV-A71's genetic diversity. Researchers must identify conserved regions or common epitopes shared among strains to neutralize a broader spectrum of variants. Extensive research is ongoing to understand the virus's genetic variability and immune responses. Efforts are being made to improve vaccine design for enhanced cross-protection against diverse EV-A71 strains [79]. Antibody-Dependent Enhancement (ADE) is a phenomenon where antibodies can enhance viral entry into host cells under certain conditions. While this process can potentially exacerbate infection in some viral diseases, it is not well-documented for EV-A71.

To mitigate the occurrence of HFMD cases, the development of a multivalent vaccine targeting multiple etiological agents is a potential approach. In addition to EV-A71, other viruses such as CVA16 are known to contribute to HFMD. By targeting these multiple viruses, the vaccine can provide broader protection against HFMD [80]. Furthermore, the development of a nasal mucosal spray vaccine can be explored to enhance the convenience and effectiveness of vaccine administration. The nasal route offers advantages in terms of improved patient compliance and increased accessibility, potentially leading to higher vaccination coverage and better control of HFMD transmission [81].

On the other hand, it is projected that the vaccination rate for EV-A71 will not come close to reaching the same level as that which was attained for poliovirus. Due to the current state of affairs, it is necessary to investigate many alternative methods, one of which is the employment of antiviral drugs as a prospective strategy. Antiviral medicines offer a compelling alternate management and treatment strategy for EV-A71 infections. Despite this, it is essential to note that there is currently no treatment in the testing phase of clinical trials that is designed to treat EV-A71 infection in particular.

Antiviral drugs that contain a wide range of activity are sought due to the commercial and practical consequences of having such agents. There is no possible way to overestimate the importance of primary research. The application of cutting-edge technology to the study of the interactions between viruses and their hosts has the potential to speed up the development of antiviral treatments that are effective against a wide variety of enteroviruses. This is one of the many potential benefits of this line of research. Viral capsid proteins have been proven to be reliable targets for antiviral drugs [82]. The high potency (nmol/L to pmol/L) of capsid inhibitors is an advantage. However, their antiviral activity is frequently restricted to particular subtypes of enteroviruses and capsid inhibitors have a low genetic barrier to drug resistance. There are currently no reported broad-spectrum capsid inhibitors [83]. However, in spite of this, capsid inhibitors exhibit considerable potential as combination therapy candidates. Additional research is required to concentrate on the enhancement of selectivity, the expansion of the antiviral spectrum, and the optimization of pharmacokinetic properties [[84], [85], [86]]. This strategy has the potential to make use of what we know about the virus and how it interacts with the host, which would make it easier to devise therapeutic strategies that are more effective. However, the interest in developing antiviral EV-A71 drugs has been diminished due to market considerations by international pharmaceutical companies. It might be worthwhile to explore the possibility of developing drugs targeting EV-A71 through local Taiwanese pharmaceutical companies or government investments. By doing so, it would be possible to focus on the regions in Asia where the virus is prevalent, thus establishing a potential market for these drugs and ultimately bridging the gap between research and clinical application.

In addition, a tantalizing prospect presents itself as a result of the recent boom in research on the microbiota of the gut. The use of probiotics, which have beneficial characteristics in boosting gastrointestinal immunity, may be employed as a preventive measure against enterovirus infection and the accompanying effects in various organs, including the brain, lungs, and heart. This is because probiotics contain favorable features in strengthening gastrointestinal immunity. This has the ability to operate as an alternative method of prevention that holds a lot of promise. The examination of the complex interaction that exists between the microbiota in the gut, the immune response, and viral infections may result in the development of novel preventative strategies that make use of the natural defenses that are already present in the human body. In the future, the utilization of Bacteroides and Clostridium genes can be extended to predict the severity of HFMD disease. It has been observed that intestinal microorganisms interact with enteroviruses, influencing the progression of HFMD. The presence of specific functions in the genes of intestinal bacteria may contribute to the development of severe symptoms in HFMD patients [87].

In conclusion, the goal of preventing and controlling EV-A71 infections is fraught with a wide variety of obstacles to overcome. In spite of this, we are in a strong position to make significant progress in this endeavor as a result of our analysis of our previous accomplishments, the adoption of stringent quality control methods, the examination of alternative methodologies, and the deployment of resources toward research endeavors. These factors have put us in a position where we are in a good position to make significant progress in this endeavor.
